# Supplementary material for: Functional ultrasound imaging combined with machine learning for whole-brain analysis of drug-induced hemodynamic changes
Source: Imaging Neurosci (Camb). 2025 Sep 10;3:IMAG.a.139. doi: 10.1162/IMAG.a.139 (PMC12423641; doi:10.1162/IMAG.a.139)
Supplement: Supplementary Material [file IMAG.a.139_supp.pdf]

Supplementary Material

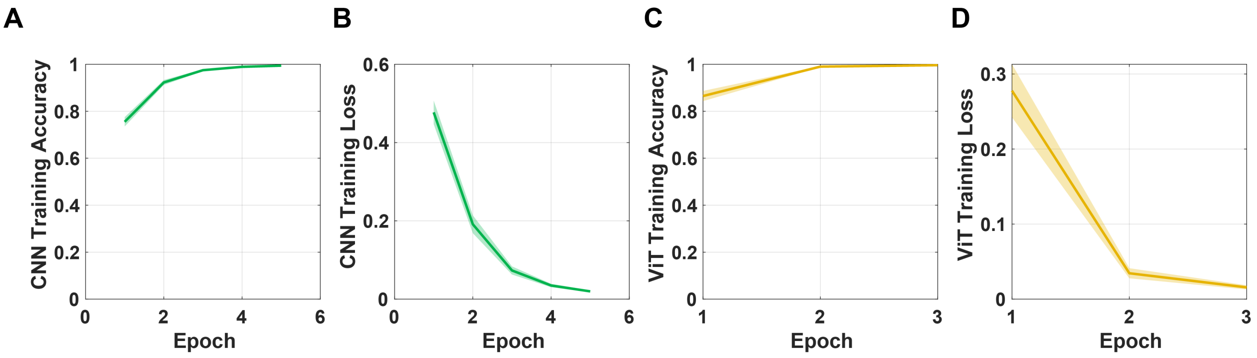

Figure S1: (A) Classification accuracy and (B) cross-entropy loss during training of CNN. (C) Classification accuracy and (D) cross-entropy loss during training of ViT. In all panels, shaded areas represent the standard error derived from averaging across cross-fold validation experiments. The CNN and ViT were trained over five and three epochs, respectively, on the final 5 minutes of post-injection fUSI data.

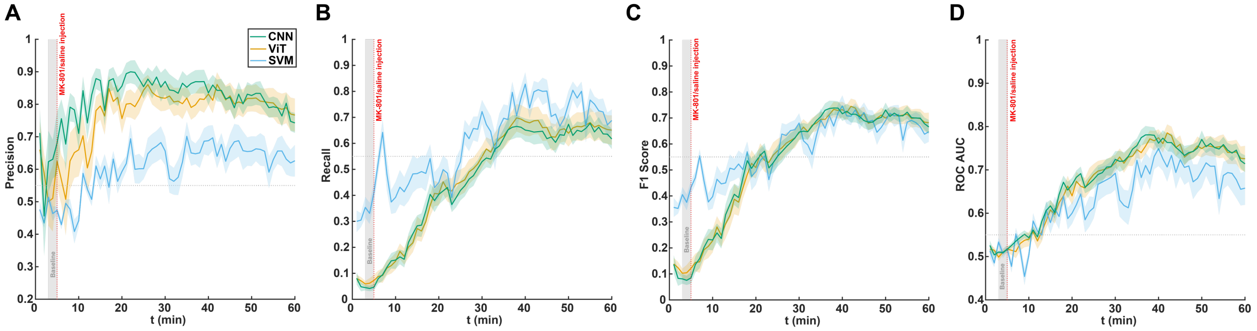

Figure S2: Additional performance metrics for distinguishing MK-801 from saline groups using CNN, SVM and ViT. (A) Precision, (B) Recall, (C) F1 Score, and (D) ROC AUC. For all panels, shaded areas represent standard error derived from averaging across animals. The vertical dotted line at 5 min marks the injection time, and the gray shaded area (minutes 3-5) indicates the baseline period.

Other supplementary materials can be found on Open Science Framework (OSF): this web link.
